# Cancer Chemotherapy: Combination with Inhibitors (Volume I)

**DOI:** 10.3390/cancers16030607

**Published:** 2024-01-31

**Authors:** Gabriella D’Orazi, Mara Cirone

**Affiliations:** 1 Unit of Cellular Networks, Department of Research, IRCCS Regina Elena Cancer National Institute, 00144 Rome, Italy; 2 Department of Neurosciences, Imaging and Clinical Sciences, University G. D’Annunzio, 00131 Chieti, Italy; 3 Department of Experimental Medicine, University of Rome La Sapienza, Viale Regina Elena 324, 00161 Rome, Italy; mara.cirone@uniroma1.it

## 1. Introduction

Cancer is one of the major causes of death globally, accounting for 10 million deaths in 2020 [1]. Despite advances in cancer treatments, including surgery, hormone therapy, gene therapy, immunotherapy, radiation therapy, laser therapy, combination therapy, and targeted therapy, conventional chemotherapy is the most common and promising treatment for cancer management [2,3,4]. However, unfortunately, the development of drug resistance often occurs, enabling cancer cells to avoid chemotherapy-induced senescence or apoptosis, leading to disease recurrence and progression [5].

Several processes have been implicated in the development of chemoresistance, particularly those able to promote adaption to proteotoxic or genotoxic stress, such as autophagy, unfolded protein response (UPR), DNA damage response (DDR), antioxidant response, and heat shock response (HSR). In this regard, recent studies have revealed that these responses are interconnected and that the inhibition of one of them can either inhibit or activate the other/s [6]. In the latter case, the concomitant inhibition of several adaptive responses is required to obtain a better therapeutic outcome compared to the inhibition of the single process, while in the former case, such cross-talk can be exploited to render cancer cells more sensitive to treatments. As an example, the inhibition of UPR can impair DDR, and this effect renders cancer cells more susceptible to treatment by DNA-damaging agents [7], and inhibition of the antioxidant response can activate the pro-survival function of UPR as a resistance mechanism [8]. Interestingly, these adaptive processes are regulated by several molecular pathways constitutively activated in cancer cells and whose interconnection may be targeted as another promising strategy to combat drug resistance. This is particularly important in the case of which the inhibition of these pathways counteracts immune suppression or even activates the anticancer immune response. Therefore, to combat drug resistance, combination therapies targeting several molecular pathways, often interconnected, seem to be more promising than a single targeted therapy.

## 2. An Overview of the Published Articles

This first volume of this Special Issue includes three original research papers and three reviews from experts in the field, providing the reader with advances in the understanding of how exploring new regimens of combination therapies may overcome chemoresistance.

For instance, in the article by Byskata and coll. [9], the authors focused on novel treatments for human papillomavirus-positive (HPV+) tonsillar and the base of tongue squamous cell carcinomas (TSCC/BOTSCC), which are rising in incidence. TSCC/BOTSCC are the two major oropharyngeal squamous cell carcinoma subtypes and have a better outcome when they are HPV+ compared with when they are HPV−, but chemoradiotherapy has not improved patient survival [10,11]. Many attempts have been made to find prognostic and therapeutical targets for this kind of tumor. Among them, the recurrence of mutations in the phosphatidyl-inositol-4,5-bisphosphate 3-kinase catalytic subunit alpha (*PIK3CA*) has been observed [12], and these mutations have sometimes been correlated to a worse outcome, so targeting this gene may be of benefit, especially upon recurrence. With this in mind, the authors of the study explored, in TSCC/BOTSCC cell lines, the effects of the PI3K inhibitor (BYL719), as well as of PARP and WEE1 inhibitors (BMN-673 and MK-1775, respectively), alone or in combinations and with or without irradiation (IR), which has been shown to enhance the effect of PARP inhibitors in other systems. They found that single PI3K, PARP, and WEE1 inhibitors significantly reduced the viability and proliferation of both HPV+/HPV− TSCC/BOTSCC cell lines in a dose-dependent manner. Interestingly, when the inhibitors were combined with 10 Gy irradiations, mainly positive/synergistic effects were obtained. However, adding 10 Gy to the already efficient positive/synergistic inhibitor combinations had no major further effects [9]. Since PI3K inhibitors have been approved for clinical use in many tumors, the results of the above study suggest that they could also be tested in TSCC/BOTSCC tumors.

Regarding PARP inhibitors (PARPis), they are small molecules currently used with success in the treatment of certain cancer patients. Their action was first shown to be specific to cells with DNA repair deficiencies, such as BRCA-mutant cancers. However, recent work has suggested clinical interest of these drugs beyond this group of patients. Thus, preclinical data have shown relationships between the activity of PARPis and other proteins involved in DNA repair. This is nicely summarized in the review by Lars Petter Jordheim [13], where the interaction of PARPis with other DNA repair proteins such as SLX4 (FANCP, BTBD12) is reported. The review hypothesizes that the possible involvement of other DNA repair mechanisms and associated PARPis activity would be of interest in the development of new therapeutic strategies based on patients’ selection or drug associations [13]. Upcoming studies into this aspect will both provide insight into the mechanism of PARPi and help to better decipher the cellular functions and networks of these proteins.

Among signaling pathways dysregulated in some cancers and involved in carcinogenesis and cancer development is AKT, which is considered to be a validated therapeutical target [14]. Thus, its inhibition may improve chemosensitivity and reduce tumor growth [15]. To add new information to AKT regulation, the study by Ye and coll. [16] analyzed the role of zinc finger protein 275 (ZNF275), a C2H2-type transcription factor, that can induce the AKT pathway in cervical cancers. The authors found that ZNF275 protein was highly expressed in cervical cancer specimens (patient-derived xenografts—PDX) compared with surrounding normal tissues. ZNF275 knockdown reduced AKT phosphorylation and induced cell death. In addition, the treatment of cervical cancer cells with the AKT inhibitor triciribine synergistically improved the cisplatin cytotoxicity, an outcome that was evident also in vivo where the cisplatin/triciribine combination regimen suppressed the growth of cervical cancer in F3-PDX mice, which was in contrast to the control group and more than the single treatments [16]. These data are in agreement with previous studies demonstrating that the AKT signaling pathway is downstream of ZNF217, and that triciribine attenuates the growth of breast cancer in xenografted mice models expressing high ZNF217 [17], strengthening the efficacy of AKT inhibition in reducing tumor growth.

Another interesting study exploring novel combination regimens is the one by Trifanescu and colleagues investigating if intrathecal Trastuzumab administration could improve oncologic outcomes in patients developing leptomeningeal metastases (LMs) from HER2-positive breast cancer [18]. Approximately 10% of HER2-positive breast cancer patients develop leptomeningeal metastases, a rapidly fatal complication; therefore, an efficient treatment option in urgently needed [19]. The results show that the intrathecal administration of Trastuzumab, systemic treatment, and radiotherapy might improve oncologic outcomes in LM HER2-positive breast cancer with manageable toxicity. Although the possible limitations of this include the small number of patients, heterogeneity of the groups, and limited access in the real world to all new drugs available in the guidelines, the results suggest that such combination treatments may positively impact the survival and quality of life of Her2-positive breast cancer patients with LM metastasis [18], making it worth further exploration.

The review by Chen summarizes preclinical and clinical studies using diverse microtubule stabilizing agents (MSA), as well as the mechanism of action, in prostate cancer [20]. In 2023, about 29% of all new cancer cases among U.S. men were estimated to be prostate cancer, and its incidence is continuously raising [21]. The androgen receptor (AR)-regulated transcriptional pathway is the main driver for prostate cancer cell growth and metastasis [22], and androgen deprivation therapy (ADT) has been a mainstay of treatments for prostate cancer since 1941. Unfortunately, the original response to ADT lasts for barely 18 to 24 months, and castration-resistant prostate cancer (CRPC) persists to progress. In this regard, other than promoting mitotic arrest, MSAs can suppress the nuclear accumulation of androgen receptor (AR), and so far, MSAs are the only chemotherapy class with significant survival benefits for patients with CRPC. The multiple mechanisms through which MSA act not only help to better map the clinical benefits of MSAs for AR-driven CRPC but also to set up a solid foundation to search for better treatments for various forms of prostate cancer [20].

Cancer fibrosis is a critical component of the tumor microenvironment (TME) which significantly impacts cancer behavior. Fibrosis can be involved in cancer initiation, growth, progression, and resistance to anticancer therapies. In addition to cancer-induced chronic inflammation as a driver of fibrosis, cancer treatments also play an important role in creating the fibrotic TME [23]. Therefore, targeting fibrosis is an appealing approach to improve cancer outcomes. Looking for novel molecular mechanisms inducing fibrosis is therefore urgent for the development of more efficient combination therapies in fibrotic cancers. In this regard, homeodomain-interacting protein kinase 2 (HIPK2), a protein kinase that controls several molecular pathways involved in cell death and development, mainly in the cancer biology field [24], has been recently highlighted also in tissue fibrosis [25]. Among the many targets regulated by HIPK2 is the onco-suppressor p53 [26]. In an in vitro study, the inhibition of HIPK2 in cancer cells has been shown to induce fibroblast differentiation into cancer-associated fibroblasts (CAFs), as well as to reduced p53 expression, leading to cancer progression and resistance to therapies [27]. These findings are in favor of a potential role for HIPK2 in limiting cancer-associated fibrosis, though further studies are required.

## 3. Conclusions

In summary, this Special Issue includes unique articles focused on novel combination therapies to overcome chemoresistance, which remains a lethal challenge in the area of cancer biology and clinics; we hope that readers enjoy reading this Special Issue. Chemoresistance-induced failure in treatment is mediated by many different molecular determinants and molecular pathways that dictate the resistance, and cancer progression in unpredictable. We hope that this Special Issue will help to further explore, in the coming years, the many molecular mechanisms involved in chemoresistance and encourage the development of new anticancer regimens.
